# Towards A Socioeconomic Model of Sleep Health among the Canadian Population: A Systematic Review of the Relationship between Age, Income, Employment, Education, Social Class, Socioeconomic Status and Sleep Disparities

**DOI:** 10.3390/ejihpe12080080

**Published:** 2022-08-16

**Authors:** F. A. Etindele Sosso, Marta Kreidlmayer, Dess Pearson, Imene Bendaoud

**Affiliations:** 1 Department on Global Health and Ecoepidemiology, Redavi Institute, Montréal, QC H4J 1C5, Canada; 2 Department of Business Administration, Alfred Nobel Business School, 8001 Zürich, Switzerland; 3 Business and Language Center, Torrens University of Australia, Adelaïde, SA 5000, Australia; 4 Clinical Pharmacology, Faculty of Medicine, University of Montreal, Montreal, QC H3T 1J4, Canada

**Keywords:** socioeconomic status, health disparities, social class, systematic review, education, income, employment, social science, sleep

## Abstract

A better understanding of the contribution of the socioeconomic status (SES) in sleep health could guide the development of population-based interventions aiming to reduce “the silent public health issue” that are sleep disturbances. PRISMA was employed to identify relevant studies having examined the association between social class, social capital, education, income/assets, occupation/employment status, neighborhood deprivation and sleep health. Sixteen cross-sectional and three longitudinal studies were selected, having sampled 226,029 participants aged from 2 months to 85 years old. Findings showed that: (1) sleep health disparities among children and adolescent are strongly correlated to parental socioeconomic indicators; (2) poor parental income, poor family SES and poor parental education are associated with higher sleep disturbances among children and adolescents; (3) lower education is a predictor of increased sleep disturbances for adults; (4) low SES is associated with high sleep disturbances in adults and old people and; (5) low income and full-time employment was significantly associated with short sleep among adults and old people. In conclusion, sleep health should be an important public health target. Such intervention would be beneficial for populational health, for all taxpayers and public administrations, which would see a reduction in absenteeism and productivity losses attributable to sleep-related health problems in the global economy.

## 1. Introduction

Recently, disparities in development and wealth have continued to grow between G20 countries and other countries across hemispheres and continents. Over past decades, the literature has documented associations between social inequalities and several cardiovascular diseases, some chronic diseases, and even neurological diseases such as sleep disorders [1]. Researchers from different backgrounds report the existence of a social gradient involved in health and variations in socioeconomic indicators, such as income, education, employment and location [2]. In short, the higher one rises in the social hierarchy, the better the health status and the lower the mortality risk. However, the association between populational sleep health and socioeconomic status (SES) remains poorly documented, despite the recent increasing importance that sleep disturbances represent for public health authorities. A Canadian study published in December 2018, conducted between 2007 and 2015 among 21,826 respondents, found a 42% increase in insomnia symptoms among Canadians aged 18 years and older [3].

SES is a marker of living conditions and habits that influence public health outcomes in a wide range, including health disparities and social inequities [4]. There is significant evidence across industrialized and developing countries showing that health status is positively associated with socioeconomic indicators such as income, education and employment [5,6,7,8]. Findings from a recent review in US child and adult populations, with data on multiple health indicators, confirmed the existence of a gradient pattern in health disparities, in a way that even groups with intermediate SES levels were consistently less healthy than those of high SES [9]. Among health disparities influenced by variations of SES, the empirical literature also demonstrated that the same socioeconomic markers are often associated with sleep disturbances.

Some epidemiological studies reported different associations between employment and sleep disturbances [10]; between education and sleep disorders [11,12,13,14]; between living conditions and individual sleep health; or behavioral risk factors of sleep disturbances [8,15,16]. Regarding ethnicity, several North American studies have shown that visible minorities have more disturbed sleep than other social groups. African American, Hispanic American and several other non-Caucasian ethnic groups had more difficulty falling asleep, had poor sleep quality and slept less than their Caucasian counterparts [17]. Thus, there appears to be a reciprocal association between the “social stress” experienced by people of certain ethnic groups, and the development of sleep disorders among their members.

Sleep health is influenced by an individual’s income because short sleep and lower sleep quality are much more prevalent among people with lower incomes [18,19]. Science from social epidemiology, sociology and sleep medicine reports a strong association between low income and poor sleep quality, as well as short sleep duration [20]. This pattern exists in all types of populations regardless of age and ethnicity, even in societies with better wealth redistribution and more egalitarian social policies, like Canada [19]. In a thorough review of published empirical research on the subject, the observed trend between income and sleep seems similar everywhere: people who are statistically, conceptually or culturally poor, sleep less and worse than people with middle or high incomes.

Furthermore, there is not enough information to understand potential pathways linking SES and sleep in general. While in recent years there has been an increasing interest in the relationship between sleep and public health [15,18,20,21,22,23,24], there is a lack of meta-analysis and systematic review on the influence of SES on sleep health in the Canadian population. By studying the SES-sleep association across a large age span, and taking into account the influence of socioeconomic indicators such as education, income and employment, it will move forward the understanding of how SES contributes to the emergence of sleep health disparities. The aims of this systematic review are: (1) to assess the association between SES and population sleep health disparities in the general Canadian population; (2) to assess if this association is similar for minors and adults and; (3) to identify potential improvements that should be tackled by federal and provincial Canadian public health authorities.

## 2. Methods

### 2.1. Literature Search

**Figure 1:**
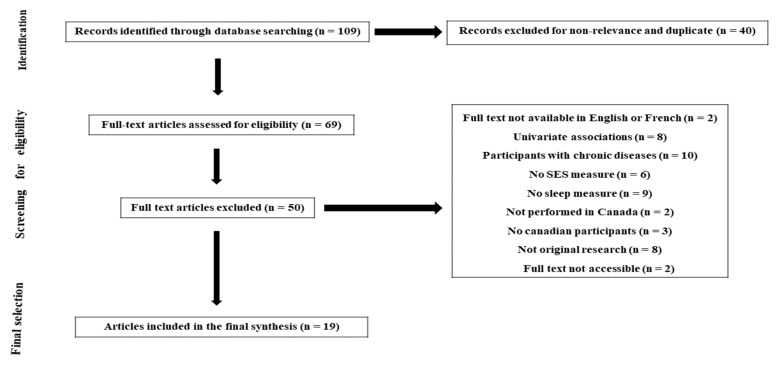
Prisma flowchart of study selection process: the relationship between SES and sleep health.

A systematic search was performed in Web of Science, Scopus, PubMed/MEDLINE and Google Scholar to identify relevant studies testing the association between SES and various sleep indicators in the Canadian population (Figure 1). The search strategy included the following terms: socioeconomic * OR socio-economic * OR “social status” OR “social position *” OR “social class *” OR “social rank *” OR education * OR income * OR occupation * OR employment OR *employed OR asset * AND sleep * OR insomnia * OR circadian OR parasomnia * OR “restless leg *” OR “periodic leg movement *” AND Canada * OR Canadian * OR Quebec * OR Ontario * OR British Columbia * OR Saskatchewan * OR Alberta * OR Manitoba * OR Nova Scotia * OR New Foundland * OR New Brunswick *. The search period included studies published from January 1990 to December 2020.

### 2.2. Inclusion Criteria

Observational studies of any design (cross-sectional, prospective or retrospective cohort) that evaluated human subjects of any age, gender or race/ethnicity from the general population were kept. Multiple objective SES measures such as education, income, occupation, employment status, assets, composite scores, subjective SES, neighborhood or residence area, deprivation indexes and self-reported perceived SES were considered. For studies examining children or adolescents, parental SES indicators such as parental education, parental occupation and household income were used instead. Objective sleep parameters, such as wrist actigraphy, accelerometry or polysomnography (PSG), and subjective reports about sleep duration, sleep quality and any symptoms of sleep complaints were all considered.

### 2.3. Exclusion Criteria

Studies were excluded for the following reasons: (1) studies were interventional trials, reviews or meta-analyses, case series or case reports and did not present original research; (2) studies were not in English or French; (3) the full text was not accessible; (4) researchers recruited participants that already presented serious health issues at baseline (i.e., cancer, diabetes, neurodegenerative diseases, mental disorders or other chronic diseases); (5) results reported univariate associations and unadjusted estimates of the variables of interest and; (6) studies were not performed in Canada, or with Canadian participants.

### 2.4. Data Extraction, Quality Assessment and Synthesis of Results

The following information was extracted from included studies: epidemiological study type, type of population, percentage of each gender, age (mean age or age range), sample size, independent variables (IV) and their measurement, sleep variables and their measurement, results and conclusions. The National Institute of Health’s Quality Assessment Tool for Observational Cohort and Cross-Sectional Studies was used to rate the quality of included studies [25]. SES was considered as the exposure, and sleep measures as the outcome variable. Studies employing subjective area SES measures, or self-reported sleep disturbances, were downgraded. Blinding of outcome assessors was non-applicable in self-reported outcomes. Studies with <50% positive rating were judged as “poor quality”, those with ≥65% as “good quality” and the rest as “fair quality”. The overall quality rating score was calculated with the proportion of positive rating on the sum of the 14 questions composing the tool and results, and are displayed in Table 1. Eligible studies were screened to extract interrater variability measurements using Cohen’s Kappa scores to assess diagnostic agreement. Interrater variability demonstrated good agreement as shown in Table S1 (Supplementary Materials).

## 3. Results

### 3.1. Characteristics of Studies

Nineteen studies [26,27,28,29,30,31,32,33,34,35,36,37,38,39,40,41,42,43,44] were included and covered the last two decades, with the oldest published in 2001 [29] and the newest in 2020 [44]. As shown in Table 2, among the 19 studies, 16 were cross-sectional studies [26,28,29,30,31,32,33,34,35,37,39,40,41,42,43,44] and three were longitudinal studies [27,36,38]. Six studies [26,35,36,38,42,43] have only children and/or adolescents as participants, whereas 12 studies [27,28,29,30,31,32,33,34,39,40,41,44] have only adult participants and one study [37] has both types of participants. Ages ranged from 2 months [43] to 85 years old [40]. Sample size ranged from 177 participants [41] to 36,984 participants [30]. The most popular socioeconomic indicators used were education [29,30,32,35,37,38,39,40,41,42,43,44], followed by income [29,30,32,33,35,36,37,38,39,40,41,42,44] and employment [29,35,37,40,41]. The main public health problems related to sleep health disparities were sleep duration [35,36,37,39,41,42,43,44], insomnia [28,29,30,33,39] and sleep quality [41,42].

### 3.2. Children and Adolescents Sleep Health Disparities

*Education:* Higher subjective SES predicted less daytime sleepiness, longer self-reported sleep duration, better sleep quality and shorter parent-reported sleep duration [42]. Sufficient sleep was more likely among students attending schools in areas classified in the highest SES group [36]. Lower SES predicted sleep rhythmic movements in children [38].

*Income:* Higher household income predicted longer parent-reported sleep duration and fewer sleep disturbances [42]. Toddlers who came from a household with a higher annual income were less likely to sleep <11 h per night [35]. Compared with children without obstructive sleep apnea (OSA), those with OSA were more likely to reside in disadvantaged neighborhoods [26]. Attending schools in low-income areas predicted short and low-normal sleep duration trajectories over time [36]. Among pre-schoolers, low household income was significantly associated with short sleep [37].

*Subjective SES and social class:* Newborns of mothers without a university degree had significantly reduced sleep duration, compared to their peers who had mothers with a university degree [43].

*Exposure and health outcome*: The main sleep health inequity investigated was sleep duration [35,36,37,42,43], followed by sleep quality [42], sleep disturbances [42] and sleep rhythmic movements [38]. Two specifics sleep disorders were studied: obstructive sleep apnea (OSA) [26] and daytime sleepiness [42]. Subjective measures were used to assess socioeconomic indicators and sleep variables, whose participants were children or adolescents in almost all studies [35,36,37,38,42,43] except one study where PSG was used to assess OSA [26]. In these studies, the mean proportion of girls is 44.9%, ranging from 26.56% [38] to 53.9% [36] of the sample. Children’s age ranged from birth [43] to 17 years old [37,42]. The sample ranged from 239 participants [42] to 36,088 participants [36].

### 3.3. Adults Sleep Health Disparities

*Education*: Overall, lower education is a predictor of increased sleep disturbances. One longitudinal investigation showed that, over a period of 10 years, prevalence of insomnia symptoms in Canada increased from 15.6% to 17.1% between 2002 and 2012, representing an absolute increase of 1.5% [28]. This increase in insomnia symptoms was significantly influenced by education [28]. Lower education was associated with more frequent insomnia symptoms [39]. People with less education were more likely to report insomnia [30]. Having some secondary (high school) education, or less, was associated with the presence of insomnia [29]. Like insomnia, a lower education level was also a risk factor of possible REM sleep behavior disorder (RBD) [40]. Finally, there is one study reporting that, among adults and older adults, less than secondary (high school) school education was significantly associated with both long sleep and short sleep [37], while another study reported that education was not significantly associated with sleep duration [44].

*Income and employment:* Overall, income and employment are strongly associated with sleep disturbances. A lower income was associated with the presence of insomnia [29]. People with lower income were more likely to report insomnia [30]. Higher annual income was associated with less sleep disturbances [33], while not enough money left over at the end of the month increased the risk of excessive daytime sleepiness (EDS) [32]. One study found that income was not significantly associated with sleep duration [44]. Not being in the work force was associated with the presence of insomnia [29]. Among older adults, full-time employment was significantly associated with short sleep, while unemployed older adults were more likely to sleep longer [37].

*Subjective SES and social class:* Overall, a low SES is associated with high sleep disturbances. A low socioeconomic status was associated with the presence of insomnia [29]. Higher SES was associated with better sleep quality, shorter sleep latency, longer sleep duration, shorter weekend oversleeps and less daytime sleepiness [41]. Subjective SES better predicted sleep duration, weekend oversleep and daytime sleepiness than objective SES, while objective SES better predicted sleep quality and latency [41]. Network capital increased the likelihood of restless sleep in men, but not women [31]. High generalized trust decreased the odds of restless sleep in women, while neighborhood disadvantages increased the odds of restless sleep in women, but not men [31]. Regardless of social class or SES, association between restless sleep, social capital and neighborhood environmental factors differed between males and females [31]. Parents with higher social capital tended to have children with fewer sleep disturbances, than parents with lower social capital [34]. One longitudinal study showed an association between incidence of subjective EDS and having a house in need of repairs [27].

*Exposure and health outcomes*: Insomnia is the most studied public health problem in sleep health disparities in Canada [28,29,30,33,39], whilst sleep duration is the second [37,39,41,44]. Sleep health disparities were also studied in EDS [27,32,41], sleep quality [41,42], sleep latency [41], RBD [40] and restless sleep [31]. Measurement of sleep variables was conducted mainly with questionnaires from the Pittsburgh Sleep Quality Index (PSQI) [41]; Epworth Sleepiness Scale (ESS) [27,32,41]; Children’s Sleep Habits Questionnaire [34]; and personalized and isolated sleep-related questions [28,29,30,31,33,37,39,40,44]. No study used actigraphy or PSG to assess sleep health outcomes. Women are more present in sleep health research in Canada, with a mean proportion of 57.75% compared to men. Regarding SES measurement, education [27,28,29,30,32,33,37,39,40,41,44] and income [27,28,29,30,32,33,37,39,40,41,44] are the most popular socioeconomic indicators used in epidemiological studies related to sleep health disparities. Employment [27,29,37,40,41], subjective SES [41] and marital status [27] are often used to assess exposure to SES influence. In addition, other proxy measures of SES, such as social capital [31,34], housing conditions [27] and neighborhood disadvantages [31] were used. All studies were cross-sectional. Sample size ranged from 177 participants [41] to 36,984 participants [30].

## 4. Discussion

### 4.1. Summary of Findings

A summarized overview of the literature shows that sleep health disparities among children and adolescents are strongly associated to parental socioeconomic indicators. Overall, poor parental income, poor family SES and poor parental education are associated with higher sleep disturbances among children and adolescents. Similar trends exist in adults’ sleep health where: (1) lower education is a predictor of increased sleep disturbances; (2) low SES is associated with high sleep disturbances and; (3) low income and full-time employment was significantly associated with short sleep. The majority of included studies [29,30,31,32,33,34,35,36,37,38,39,40] were rated as “fair quality”, while two longitudinal studies [27,38] and the single study using PSG to assess sleep health [26] were rated as “good quality”.

**Figure 2:**
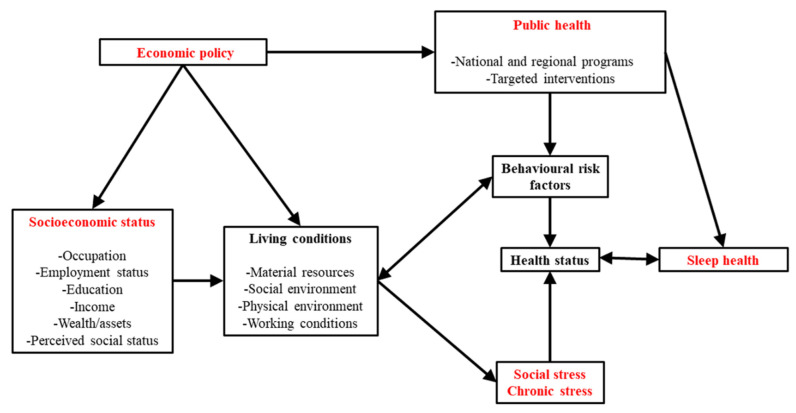
The socioeconomic model of sleep health.

An overview of the current findings confirms an underlying relationship between SES and sleep health disparities. Regardless of the sleep disturbances investigated, socioeconomic inequities appear in the sleep health of the general population, similar to what has been published in health disparities related to cardiovascular diseases, mental disorders and several chronic diseases [6,7,45,46]. While epidemiological data available on sleep medicine focused on a variety of parameters, such as sleep quality, sleep duration or sleep efficiency; the concept of ‘‘Sleep health’’ for public health is more mitigated because it is more multidimensional [31,44,47,48,49,50]. Figure 2 represents a socioeconomic model of sleep health with multiple interactions at both individual, community and governmental levels.

### 4.2. The Socioeconomical Model of Sleep Health

A detailed analysis of literature confirms a relationship between individual socioeconomic status and sleep health disparities. Regardless of which sleep disturbances are studied, socioeconomic disparities appear in the sleep health of the general Canadian population similar to what has been published in health disparities related to cardiovascular diseases, mental disorders and several chronic diseases; both in Canada and in other countries. While epidemiological data available on sleep medicine focused on a variety of parameters such as sleep quality, sleep duration or sleep efficiency; the concept of ‘‘Sleep health’’ for public health is more mitigated because it is more multidimensional and relatively new. Figure 2 presents a socioeconomical model of sleep health with multiple interactions at both individual, community and governmental levels. It will allow a mapping of socioeconomic and biobehavioral determinants, global patterns and public health trends related to sleep health in Canada, as well as other countries.

#### 4.2.1. The Economic Policy

Worldwide, the shift between our lifestyles and the solar cycle is reaching epidemic proportions. It would seem that modern life is increasingly incompatible with adequate/sufficient sleep [51]. It is, therefore, no coincidence that the countries most affected by the “silent epidemic of sleep disorders” are either G20 countries (e.g., India and South Africa) or fast-growing countries (e.g., Indonesia) [48]. The economic costs caused by insufficient sleep are significant, especially in G7 countries: the United States suffers by far the biggest economic losses (up to US$411 billion per year) due to the size of its economy, followed by Japan (up to US$138 billion per year) [51]. On an annual basis, the United States loses the equivalent of about 1.23 million working days due to insufficient sleep; followed by Japan, with an average of 604,000 working days per year; the United Kingdom and Germany with 207,000 and 209,000 days, respectively; and finally, Canada, with about 78,000 working days lost [51]. Sleep thus seems incompatible with the endless capitalist imperatives of productivity and consumption that impose longer working days, at the expense of mental health and sleep. The mechanisms for this forced interference of the western lifestyle in health status are numerous: night work, longer commute times due to the urban sprawl, new wireless technologies and more recently, teleworking. All these environmental factors have a direct or indirect association with ”sleep disorders”, knowing that a sleep of less than 6 h per night affects all the functions of an organism (cellular rest, protein synthesis, mood regulation, memory capacity) [52,53,54,55].

On the other hand, political authorities, in coordination with public health managers, employers’ associations and trade unions should redouble their efforts to align workers’ conditions with their mental health [5]. Not only would such an intervention be beneficial for the sleep of individuals, and thus for the overall health of the population, but it could also be beneficial to companies and public administrations, which would see a reduction in absenteeism and productivity losses attributable to sleep disturbances in their employees [56]. Indirectly, all taxpayers would save money due to the reduction of sleep-related health problems.

#### 4.2.2. The Individual, Family and Community SES

SES acts as a marker of the conditions of various environmental factors, such as the neighborhood [8], the social environment [6,57], work [46,58] and access to material resources [8,21,59,60]. Associations between socioenvironmental factors and multiple health outcomes are well established, suggesting a positive association between the SES and exposure to health-promoting socioenvironmental factors [7,61,62,63,64,65].

Recent meta-analyses [66,67,68] and systematic reviews [69,70] show that the relation between SES indicators (such as education, income and employment) and sleep health may be modified by age, gender and ethnicity; but it can also be mediated by factors related to living conditions, behaviour and stress [8,18]. Age, sex/gender and race/ethnicity are the main moderators of the relation between SES and sleep health, with higher rates of self-reported sleep disturbances among non-Caucasian relative to Caucasian individuals [6,22,23,71] and an increase in sleep disturbances among old people compared to young adults and children [15,23,72,73,74]. Black young adults reported shorter sleep duration as well as Hispanic young adults, and both ethnic group members slept less than their young Caucasian peers. Thus, these three moderators could be involved in a triple interaction [22,57,74,75].

#### 4.2.3. The Living Conditions and Behavioral Risk Factors 

The relation between living conditions and behavioral risk factors contributes to sleep health outcomes and influences chronic stress [38,72,76]. An irregular bedtime schedule due to working conditions (like rotative shift work and night shift work) and lack of public transportation, affect the circadian rhythm and sleep duration, leading to sleep disturbances like poor sleep quality and sleepiness [77,78,79,80]. Lifestyle factors such as smoking, drug use or unhealthy eating (the prevalence of which is associated with SES), are also associated with higher odds of being affected by sleep disturbances and an increase in both physical and chronic ailments [80,81]. Certain characteristics of a neighborhood, such as accessibility to green spaces, shops, presence of sufficient lighting or proximity to conveniences stores (restaurant, pharmacy, delivery services) and healthcare services are determinants of important healthy behaviors, including regular sleeping hygiene [41,82,83,84]. Inadequate living conditions were found to be associated with mental disorders and chronic stress in several studies [4,23,61,64,74]. The chronic stress itself is associated with several health outcomes such as cardiovascular disease [16,63,85], mental health disorders [58,61,86,87] and sleep health [73,86].

#### 4.2.4. Chronic Stress and Social Stress

Regarding chronic stress, its consequences are numerous and the links to social stress through living conditions, influence of SES and its moderators such as ethnicity and sex/gender, are manifold [6,8]. Neighborhood characteristics have been associated with high levels of stress among individuals living in noisy, unsafe neighborhoods, or ones with poor access to public transportation, parks or grocery stores [6,8,15,73]. A work environment with changing or irregular schedules contributes to the social isolation of the individual, while stressing the body with a disruption of the biological clock [5]. Psychological stress from work impacts the individual’s mental well-being and exposes them to mental health problems [5,46]. A family’s material deprivation can lead to chronic stress (i.e., late bills, poor quality housing), leading to further health problems [6,8,61]. Exposure to this social stress over time leads to chronic stress and subsequently to an increase in the allostatic load (AL) [88,89]. AL represents the physiological burden the body experiences when repeated responses to stressors are activated constantly, or for an unusually long period [85]. This chronic stress is associated with neuropsychiatric disorders such as affective and mood disorders, which are both associated with sleep health [90,91]. Chronic stress can adversely affect sleep quality and sleep duration, while insufficient sleep can increase stress levels [61,86]. In addition, both stress (chronic and social) and lack of sleep can lead to lasting physical and mental health problems [86]. Considering previous studies and reviews reporting on associations between SES and chronic stress [8,87], and other studies linking chronic stress and sleep health [23,61,92,93], the present conceptual socioeconomic model assumed that SES influences stress through living conditions, and this stress in turn influences sleep health as well as other physical and mental health disorders [15,57]. While acutely adaptive, brain reactivity and body resilience associated with chronic stress are sharply influenced by individual differences in behavioral risk factors [61] and living conditions [4,65,94,95,96].

#### 4.2.5. Sleep Disturbances

This term includes: (1) subjective sleep complaints reported by participants themselves, or measured with validated or customized questionnaires/items such as the Pittsburgh Sleep Quality Index or Insomnia Severity Index and; (2) objective “sleep disorders” confirmed with a quantitative measure of sleep complaints, such as actigraphy and PSG, or confirmed by a physician’s diagnosis or a certified sleep specialist. Sleep disturbances are associated with anxiety, depression and stress [86]; most of these diseases (including sleep disturbances) are prevalent among low SES individuals [18,42,61,86,87,95,97,98,99]. Knowing that chronic stress, social stress and AL are well known determinants of mental disorders [6,23] and difficult living conditions induced chronic stress [6,18,23], there is no doubt that sleep disturbances are consequences of these interactions [18,42,61,86,87,95,97,98,99].

### 4.3. Implications for Public Health Policy

Living conditions, behavioral risk factors and health status can all be found in the pathway from SES to sleep health inequalities (Figure 2). As such, clinical guidelines should consider them in the prevention and management of “sleep disorders”, due to their multidimensional impacts on sleep. Since adopting and maintaining good sleep hygiene is a key non-pharmacological strategy for improving the mental health and general well-being of individuals (along with modification of other behavioral risk factors such as quitting smoking or engaging in physical activity), clinicians should, therefore, tailor their interventions to the specific needs of their patients. Clinicians should consider the SES of individuals as a marker of increased risk for sleep health disparities, but should also be aware that the negative impact of SES on risk of sleep disturbances varies according to individual characteristics (like age, gender/sex, ethnicity/race). Many studies reported lower rates of neuropsychiatric disorders and other chronic comorbidities among good sleepers, compared to people suffering from sleep disturbances [16,63,92,93,100], suggesting that good sleep quality and appropriate sleep duration may protect against some mental disorders [16,63,92,93,100]. At the same time, several studies showed that a majority of good sleepers are people with high SES under good living conditions [1,21,23,59,74,98,101,102,103,104] and mental disorders (in addition to sleep disturbances) are very often reported among vulnerable populations with low SES [16,23,105]. This evidence together reveals that the protective factor that sleep can have on health is unequally distributed among different social classes and contributes to social inequalities in sleep health [23,59,73,105]. Thus, consideration of SES in sleep health management also applies to public health decision-makers and governments, who can take action through targeted interventions that support the development of healthy sleep environments.

### 4.4. Implications for Future Research

Future research should be careful in the use of the terms SES and social position. The vast majority of studies in sleep medicine unfortunately seem to equate social position with living conditions, leading to possible confusion as to what is really impacting health in general, and sleep health in particular. The present conceptual socioeconomic model clarifies the distinction between these two concepts and provides tips on which is more suitable for the research question. Researchers from biomedical fields, and applied sciences in particular, should think deeply about how to measure and integrate social inequalities in health into their experimental designs. Sleep health seems to be a common factor in many health outcomes [67,68,106] and it would be important to design new instruments or to update current questionnaires of self-reported sleep outcomes to include, as standard basic items, questions or variables related to SES, social position and living conditions.

### 4.5. Current Limitations and Challenges

The first challenge is the diversity of SES measures in the literature. The variety of definitions and conceptualizations of SES leads to a substantial heterogeneity in the measurements of SES. Nevertheless, considering that SES is an emerging concept, such diversity contributes to validating the role of SES in sleep health disparities, as multiple studies reported results pointing in the same direction. The second important challenge is how sleep health is measured. Several studies assessed sleep health with a single question, an approach that cannot capture the multi-dimensionality and day-to-day variability of sleep, and may be unable to detect the accurate effect SES has on sleep health. Furthermore, indicators were obtained for most of the studies through participants’ self-report, which could mean that recall bias might have altered the accuracy of the findings. Effects of SES variations on objective sleep measures, like actigraphy and PSG, are still unknown. Investigating the association SES-sleep health has with objective measurement will certainly bring more knowledge to the entire scientific community and decision-makers, and it will also help to update this socioeconomic model of sleep health. Finally, another important challenge is the experimental setting of the study. The cross-sectional design employed by most studies limits and restricts conclusions about causality. It seems redundant to say, however, a reminder is of utmost importance. Longitudinal studies have the advantage to follow changes through time, which is a game changer for advancement of knowledge, especially with a complex biological function like sleep, in which physiological and behavioral parameters change a lot from youth to adulthood.

## 5. Conclusions

Sleep has been studied intensively in the past two decades and remains a subject of interest, overlapping several disciplines such as psychology, neurology, pneumology and psychiatry [107]. Incidence and prevalence of sleep disturbances have increased globally over the past decade [30,108,109,110,111,112,113], while their determinants, as well as associated psychopathological mechanisms, remain not well understood. More than any other external factors, the interaction between SES and sleep health must be better understood. Future work should identify how factors related to living conditions and lifestyle/habits act on the incidence and progression of sleep health disparities, not only in the Canadian population, but worldwide. This socioeconomic model of sleep makes it possible to identify potentially vulnerable populations (i.e., low-income people, children living in disadvantaged families, individuals with limited education, people living in impoverished neighborhoods) for whom specific interventions could be developed. Public health interventions aimed at improving living conditions and reducing social inequalities are likely to contribute to the improvement of sleep health, helping at the same time in reducing chronic and social stress of low SES populations. Some examples of efficient interventions would include the reduction of rotative shift work, an increase in availability and accessibility to neighborhood green spaces and a massive promotion of good sleep health.

## Figures and Tables

**Table 1:** Quality assessment of included studies according to National Institute of Health’s Quality Assessment Tool for Observational Cohort and Cross-Sectional Studies.

Author’s Name & Year	Q1	Q2	Q3	Q4	Q5	Q6	Q7	Q8	Q9	Q10	Q11	Q12	Q13	Q14	Quality Score
**Brouillette 2011 [26]**	Y	Y	Y	Y	Y	N	Y	Y	Y	N	N	Y	Y	Y	**Good**
**Karunanayake 2018 [27]**	Y	Y	Y	Y	N	N	Y	N	Y	N	N	Y	Y	Y	**Good**
**Garland 2018 [28]**	Y	Y	Y	Y	Y	N	Y	Y	Y	N	N	Y	Y	Y	**Good**
**Sutton 2001 [29]**	Y	Y	Y	Y	N	N	N	Y	Y	N	N	NA	NA	Y	**Fair**
**Tjepkema 2005 [30]**	Y	Y	Y	Y	N	N	N	Y	Y	N	N	NA	NA	Y	**Fair**
**Bassett 2014 [31]**	Y	Y	Y	Y	N	Y	Y	Y	N	N	N	NA	N	Y	**Fair**
**Gjevre 2014 [32]**	Y	Y	N	Y	N	N	N	Y	Y	N	N	NA	NA	Y	**Fair**
**Baiden 2015 [33]**	Y	Y	NR	Y	N	N	N	Y	Y	N	N	NA	NA	Y	**Fair**
**Nagy 2016 [34]**	Y	Y	NR	Y	N	Y	Y	N	Y	N	N	NA	N	Y	**Fair**
**Costanian 2017 [35]**	Y	Y	Y	Y	N	N	N	Y	Y	N	N	NA	NA	Y	**Fair**
**Patte 2017 [36]**	Y	Y	Y	Y	N	Y	Y	Y	N	N	N	NA	N	Y	**Fair**
**Chang 2018 [37]**	Y	Y	Y	Y	N	N	N	Y	Y	N	N	NA	NA	Y	**Fair**
**Laganiere 2019 [38]**	Y	Y	NR	Y	N	Y	Y	N	Y	N	N	NA	N	Y	**Fair**
**Vézina-Im 2019 [39]**	Y	Y	Y	Y	N	N	N	Y	Y	N	N	NA	NA	Y	**Fair**
**Yao 2019 [40]**	Y	Y	Y	Y	N	N	N	Y	Y	N	N	NA	NA	Y	**Fair**
**Jarrin 2013 [41]**	Y	N	NA	Y	N	N	N	Y	Y	N	N	NA	NA	Y	**Poor**
**Jarrin 2014 [42]**	Y	N	NA	Y	N	N	N	Y	Y	N	N	NA	NA	Y	**Poor**
**Matenchuk 2019 [43]**	Y	Y	NR	Y	N	N	N	N	Y	N	N	NA	NA	Y	**Poor**
**Seaton 2020 [44]**	Y	Y	NR	Y	N	N	N	N	Y	N	N	NA	NA	Y	**Poor**

Y = Yes; N = No; NA = not applicable; NR = not reported.

**Table 2:** Characteristics of epidemiological studies investigating sleep disparities in the Canadian population.

**Studies Including Only Children and/or Adolescents < 18 Years Old**										
**Study**	**Study Type**	**Type of Population**	**% Women**	**Mean Age or Age Range (years)**	**Sample** **Size (n)**	**Exposure**	**Exposure Measurement**	**Health Outcome**	**Health Outcome Measurement**	**Results and Conclusions**
**Brouillette 2011 [26]**	Cross-sectional	Children from Montréal	41	2–8	436	Neighbourhood SES	Neighborhood characteristics were evaluated at the census tract level based on the 2006 Canadian census.	Obstructive Sleep Apnea (OSA)	PSG	Compared with the children without OSA, those with OSA were more likely to reside in disadvantaged neighbourhoods
**Jarrin 2014 [42]**	Cross-sectional	Children and adolescents recruited from schools and neighbourhoods in Montreal	45.6	8–17	239	(a) Income (b) Education (c) Social class	(a) Household income divided into 17 categories (b) Highest parental education divided into 9 categories (c) Subjective Social Status Scale-Youth Version (two 10-rung ladders: school and society, youth reported)	(a) Sleep quality (b) Daytime sleepiness (c) Sleep disturbances (d) Sleep duration	(a) youth-rated 10-point scale (b) Pediatric Daytime Sleepiness Scale (c) Children’s Sleep Habits Questionnaire (d) Self-reported sleep duration	In children, higher subjective SES predicted less daytime sleepiness and longer self-reported sleep duration and higher household income predicted longer parent-reported sleep duration. In adolescents, higher subjective SES was associated with better sleep quality and shorter parent-reported sleep duration, and higher household income was associated with fewer sleep disturbances.
**Patte 2017 [36]**	(a) Cross-sectional (b) Longitudinal cohort followed for 2 years	Adolescents 9th–12th grade from secondary schools in Ontario and Alberta	53.9	6–14	(a) 36,088 (b) 7394	Income	School area average income (median household income of census divisions that corresponded with school postal codes according to data from the 2011 National Household Survey)	Sleep duration	(a) Short sleep duration (<8 h) (b) Sleep duration trajectories (short, low-normal, high-normal, long)	(a) Sufficient sleep was more likely among students attending schools in areas classified in the highest SES group (b) Attending schools in low-income areas predicted short and low-normal sleep duration trajectories over time
**Costanian 2018 [35]**	Cross-sectional	Toddlers from the general population	49.6	1–2	3675	(a) Income (b) Employment status (c) Education	(a) Household income (<$30,000, $30,000–<$60,000, $60,000–<$100,000, ≥$100,000) (b) Mother’s work status (currently working vs. not currently working) (c) Mother’s education level (college graduate or less vs. more than college graduate)	Sleep duration	Parent-reported sleep duration (<11 h vs. more)	Toddlers who came from a household with higher annual income were less likely to sleep <11 h per night
**Chang 2018 [37]**	Cross-sectional	Participants from the general population	48.13	3–17	4924	(a) Education (b) Income (c) Employment	(a) Highest level of education attained in the household for pre-schoolers, children and youth or by the respondent for adults and older adults (less than secondary school degree, secondary school degree, postsecondary school degree) (b) Household income adequacy (based on total annual household income and number of people living in the household and categorized as low, middle, or high) (c) Employment status (full-time, part-time, unemployed) for adults and older adults	Sleep duration	Self-reported or parent-reported (when participant have less than 12 years) sleep duration (recommended, short, long according to guidelines)	Among pre-schoolers, low household income was significantly associated with short sleep.
**Laganiere 2019 [38]**	Longitudinal cohort followed for 4 years	Children recruited at birth in obstetric clinics of Montreal and Hamilton	26.56	0–4	529	Global SES estimated with (a) education and (b) income	High SES (high maternal education level and high income) vs. middle/low SES (low on at least one of the variables)	Sleep rhythmic movements	Single question + Children ’s Sleep Habits Questionnaire)	Lower SES predicted sleep rhythmic movements in children
**Matenchuk 2019 [43]**	Cross-sectional	Newborn of a population-based birth cohort in Edmonton	49.56	0.25	619	Education	Maternal education (university degree vs. lower)	Sleep duration	Parent-reported sleep duration	Newborns of mothers without a university degree had significantly reduced sleep duration compared to those of mothers with a university degree
**Studies Including Participants with All Ages**										
**Study**	**Study Type**	**Type of Population**	**% Women**	**Mean or Range Age (years)**	**Sample** **Size (n)**	**Exposure**	**Exposure Measurement**	**Health Outcome**	**Health Outcome Measurement**	**Results and Conclusions**
**Sutton 2001 [29]**	Cross-sectional	Adults from the general population	NA	≥15	10,702	(a) Education (b) Income (c) Employment	(a) Scale items are: some secondary or less, secondary graduation, some post-secondary, post-secondary degree or diploma (b) Income adequacy (lowest, next to lowest, middle, next to highest or highest) (c) working status (not in the work force, usually workdays, regular shift work)	Insomnia	Single question (yes vs. no)	Low socioeconomic status, reflected by having some secondary education or less, lowest income and not being in the work force, was associated with the presence of insomnia
**Tjepkema 2005 [30]**	Cross-sectional	Adults from the general population	NA	≥15	36,984	(a) Education (b) Income	(a) Scale items are: less than secondary graduation, secondary graduation, some postsecondary until postsecondary graduation (b) Household income (lowest, lower-middle, upper-middle, highest)	Insomnia	Insomnia symptoms frequency (none of the time, a little of the time, or some of the time vs. most of the time or all of the time)	People with less education and lower income were more likely to report insomnia
**Jarrin 2013 [41]**	Cross-sectional	Adults from advertisements in Montreal	81.4	30–65	177	(a) Income (b) Education (c) Employment (d) Subjective SES	(a) Household income (b) Years of education (c) Employment status (employed vs. unemployed) (d) MacArthur Scale of Subjective Social Status (scale 1–10)	(a) Sleep quality (b) Sleep latency (c) Sleep duration (d) Weekend oversleep (e) Daytime sleepiness	(a) Sleep quality (PSQI Global score) (b) Sleep latency (PSQI sleep latency subscale) (c) Weekday sleep duration (d) Difference between weekend and weekday total sleep duration (e) Measured with Epworth Sleepiness Scale (ESS)	Higher SES was associated with better sleep quality, shorter sleep latency, longer sleep duration, shorter weekend oversleeps and less daytime sleepiness. Subjective SES better predicted sleep duration, weekend oversleep and daytime sleepiness than objective SES. Objective SES better predicted sleep quality and latency than subjective SES.
**Bassett 2014 [31]**	Retrospective cross-sectional	Adults from the 2008 Montreal Neighborhood Networks and Healthy Aging Study	64.81	≥25	2643	(a) Neighborhood disadvantage (b) Social capital (c) SES	(a) Neighbourhood disadvantage measure was created using six census tract variables: unemployment rates, median household income, the percentage of immigrants, the percentage of single mothers, the percentage of renters, and the percentage of college educated residents (b) Social capital was measured with the network capital, generalized trust and neighborhood volunteering (c) SES score was created using principal components analysis of respondents’ income, education, and employment status	Restless sleep	Participants responded yes or no to the item “my sleep was restless.” extracted from the Center for Epidemiologic Studies Depression (CES-D) scale	Women were more likely to experience restless sleep than men. Network capital increased the likelihood of restless sleep in men but not women. High generalized trust decreased the odds of restless sleep in women. Neighbourhood disadvantages increased the odds of restless sleep in women but not men. The association among restless sleep, social capital, and neighbourhood environmental factors differed in male and female Montreal adults.
**Gjevre 2014 [32]**	Cross-sectional	Adults from the general population in Saskatchewan	50.8	>18	7597	(a) Income (b) Education	(a) Household income adequacy (4 levels) Money left over at the end of the month (some, just enough, not enough) (b) Items used are: less than high school, completed high school, completed university, completed other postsecondary education	Excessive Daytime Sleepiness (EDS)	ESS score >10	Not enough money left over at the end of the month increased the risk of EDS
**Baiden 2015 [33]**	Cross-sectional	Participants from the general population	49.36	>20	19,349	(a) Education (b) Income	(a) Postsecondary education (no vs. yes) (b) Annual personal income (6 levels)	Insomnia	Insomnia symptoms (most/all of the time vs. none/a little of/some of the time)	Higher annual income was associated with less sleep disturbances
**Nagy 2016 [34]**	Cross-sectional	Parents with child of brain-to-society study in Montréal	73.1	41.75	339	(a) Parental social capital (b) Income	Position generator	Child sleep disturbances	Children’s Sleep Habits Questionnaire	Parents with higher social capital tended to have children with fewer total sleep disturbances than did parents with lower social capital
**Chang 2018 [37]**	Cross-sectional	Participants from the general population	51.45	18–79	7250	(a) Education (b) Income (c) Employment	(a) Highest level of education attained in the household for pre-schoolers, children and youth or by the respondent for adults and older adults (less than secondary school degree, secondary school degree, postsecondary school degree) (b) Household income adequacy (based on total annual household income and number of people living in the household and categorized as low, middle, or high) (c) Employment status (full-time, part-time, unemployed) for adults and older adults	Sleep duration	Self-reported or parent-reported (when participant have less than 12 years) sleep duration (recommended, short, long according to guidelines)	Among older adults, less than secondary school education and full-time employment were significantly associated with short sleep. Among adults and older adults, less than secondary school education was significantly associated with long sleep. Unemployed older adults were more likely to sleep longer.
**Garland 2018 [28]**	Retrospective cross-sectional	Adults from the general canadian population	55	≥20	34,118 in 2002 And 23,089 in 2012	Education	Secondary analysis of Data from the Canadian Community Health Survey-Mental Health cycles 2000–2002 and 2011–2012	Insomnia	The question “How often do you have trouble going to sleep or staying asleep?”	Over a 10-year period, prevalence of insomnia symptoms increased from 15.6% to 17.1% between 2002 and 2012, representing an absolute increase of 1.5%. The likelihood of occurrence of insomnia symptoms was significantly influenced by education
**Karunanayake 2018 [27]**	Longitudinal	Adults from Canadian indigenous populations in Saskatchewan	52.4	≥18	317	(a) Income (b) Housing conditions (c) Employment status (d) Education (e) Marital status	Secondary analysis of Data from the First Nations Lung Health Project (FNLHP)	Excessive daytime sleepiness (EDS)	Epworth Sleepiness Scale (ESS)	This study showed an association between incidence of subjective EDS and less money left over at end of the month and having a house in need of repairs
**Yao 2018 [40]**	Cross-sectional	Adults from the general population	56.91	45–85	19,584	(a) Education (b) Income (c) Employment	(a) Education (middle school and under, secondary school, bachelor’s degree and above) (b) Annual personal income (<$20,000, $20,000–$49,000, $50,000–$99,000, ≥$100,000) (c) Employment status (employed vs. retired)	Possible RBD	Single question (yes vs. no)	Lower education level was a risk factor of possible RBD
**Vézina-Im 2019 [39]**	Cross-sectional	Women from the general population	100	18–44	9749	(a) Education (b) Income	(a) Items used are: less than high school; high school diploma; some postsecondary studies; postsecondary certificate/diploma or university degree (b) Household income	(a) Sleep duration (b) Insomnia	(a) Insufficient sleep duration (<7 h) (b) Insomnia symptoms (none/little of the time vs. some/most/all the time)	Lower education was associated with more frequent insomnia symptoms
**Seaton 2020 [44]**	Cross-sectional	Male employees from six workplaces in northern British Columbia	0	18–66	227	(a) Education (b) Income	(a) Items used are: some high school, completed high school, trades certification/college diploma, university degree (b) Items used are: >CAD $100,000, CAD $50,000–CAD $100,000, <CAD $50,000)	Sleep duration	Self-reported sleep duration	Education and income were not significantly associated with sleep duration

EDS = excessive daytime sleepiness; ESS = Epworth Sleepiness Scale; NA = Not Available; OSA = Obstructive Sleep Apnea; PSQI = Pittsburgh Sleep Quality Index; REM = rapid eye movement; RBD = REM sleep behaviour disorder; SES = socioeconomic status.

## Data Availability

See Table S1.

## Supplementary Materials

The following supporting information can be downloaded at: https://www.mdpi.com/article/10.3390/ejihpe12080080/s1, Table S1.
