# Supplementary material for: Towards A Socioeconomic Model of Sleep Health among the Canadian Population: A Systematic Review of the Relationship between Age, Income, Employment, Education, Social Class, Socioeconomic Status and Sleep Disparities
Source: Eur J Investig Health Psychol Educ. 2022 Aug 16;12(8):1143–67. doi: 10.3390/ejihpe12080080 (PMC9407487; doi:10.3390/ejihpe12080080)
Supplement: Supplementary file 1 [file ejihpe-12-00080-s001.zip › ejihpe-1723295-supplementary.pdf]

Article number	Included (Y) & Excluded (N)	Kappa
1	N	0,63
2	N	0,65
3	Y	0,82
4	N	0,6
5	Y	0,84
6	Y	0,75
7	Y	0,81
8	Y	0,86
9	N	0,67
10	N	0,63
11	N	0,69
12	Y	0,8
13	N	0,64
14	N	0,67
15	Y	0,85
16	Y	0,89
17	Y	0,8
18	N	0,7
19	Y	0,64
20	Y	0,64
21	N	0,69
22	N	0,68
23	N	0,71
24	N	0,66
25	N	0,64
26	N	0,72
27	N	0,65
28	N	0,63
29	N	0,68
30	N	0,65
31	N	0,66
32	Y	0,82
33	N	0,63
34	N	0,68
35	N	0,65
36	N	0,63
37	N	0,75
38	Y	0,82
39	N	0,64
40	N	0,65
41	Y	0,86
42	N	0,64
43	Y	0,81
44	Y	0,68

45	N	0,74
46	N	0,76
47	N	0,72
48	N	0,72
49	N	0,65
50	N	0,74
51	N	0,68
52	N	0,63
53	N	0,7
54	N	0,7
55	N	0,68
56	N	0,64
57	N	0,76
58	N	0,63
59	N	0,72
60	N	0,68
61	N	0,64
62	N	0,7
63	N	0,72
64	N	0,66
65	N	0,74
66	Y	0,82
67	N	0,63
68	N	0,66
69	N	0,62
70	N	0,74
71	N	0,65
72	N	0,72
73	N	0,73
74	N	0,76
75	N	0,65
76	N	0,63
77	N	0,67
78	N	0,64
79	N	0,72
80	N	0,76
81	N	0,74
82	N	0,71
83	Y	0,87
84	N	0,74
85	N	0,64
86	N	0,74
87	N	0,69
88	N	0,72
89	N	0,71
90	N	0,74

91	N	0,67
92	Y	0,82
93	N	0,67
94	N	0,64
95	Y	0,89
96	N	0,65
97	N	0,67
98	N	0,74
99	N	0,76
100	N	0,68
101	Y	0,85
102	N	0,74
103	N	0,67
104	N	0,64
105	N	0,72
106	N	0,74
107	N	0,76
108	N	0,65
109	Y	0,86
